# 4-Meth­oxy-2-nitro-4′-(trifluoro­meth­yl)biphen­yl

**DOI:** 10.1107/S1600536811039092

**Published:** 2011-10-12

**Authors:** Yan-Jun Hou, Xin-Min Li, Wen-Yi Chu, Zhi-Zhong Sun

**Affiliations:** a College of Chemistry and Materials Science, Heilongjiang University, Harbin 150080, People’s Republic of China

## Abstract

The title compound, C_14_H_10_F_3_NO_3_, was prepared by a palladium-catalysed Suzuki–Miyaura coupling reaction. The dihedral angle between the nitro group and its parent benzene ring is 66.85 (19)° while the dihedral angle between the two benzene rings is 49.98 (9)°. The CF_3_ group is disordered over two sets of sites with occupancies of 0.457 (8) and 0.543 (8).

## Related literature

For general background to the synthesis and properties of the title compound, see: Suzuki (1999 ▶); Razler *et al.* (2009 ▶). For the biological activity of biphenyl derivatives, see: Kimpe *et al.* (1996 ▶).
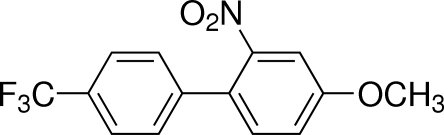


## Experimental

### 

#### Crystal data

C_14_H_10_F_3_NO_3_
*M* *_r_* = 297.23
Monoclinic, 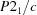

*a* = 8.1956 (13) Å
*b* = 20.777 (3) Å
*c* = 7.9715 (12) Å
β = 104.240 (2)°
*V* = 1315.7 (3) Å^3^
*Z* = 4
Mo *K*α radiation
μ = 0.13 mm^−1^
*T* = 293 K
0.26 × 0.24 × 0.20 mm

#### Data collection

Bruker APEXII CCD area-detector diffractometer
Absorption correction: multi-scan (*SADABS*; Sheldrick, 1996 ▶) *T*_min_ = 0.966, *T*_max_ = 0.974
10512 measured reflections
3235 independent reflections
1910 reflections with *I* > 2σ(*I*)
*R*_int_ = 0.029

#### Refinement

*R*[*F* ^2^ > 2σ(*F* ^2^)] = 0.050
*wR*(*F* ^2^) = 0.161
*S* = 1.05
3235 reflections
243 parameters
36 restraints
H atoms treated by a mixture of independent and constrained refinement
Δρ_max_ = 0.30 e Å^−3^
Δρ_min_ = −0.21 e Å^−3^

### 

Data collection: *APEX2* (Bruker, 2004 ▶); cell refinement: *SAINT* (Bruker, 2004 ▶); data reduction: *SAINT*; program(s) used to solve structure: *SHELXS97* (Sheldrick, 2008 ▶); program(s) used to refine structure: *SHELXL97* (Sheldrick, 2008 ▶); molecular graphics: *SHELXTL* (Sheldrick, 2008 ▶); software used to prepare material for publication: *publCIF* (Westrip, 2010 ▶).

## Supplementary Material

Crystal structure: contains datablock(s) I, global. DOI: 10.1107/S1600536811039092/ff2027sup1.cif

Structure factors: contains datablock(s) I. DOI: 10.1107/S1600536811039092/ff2027Isup2.hkl

Supplementary material file. DOI: 10.1107/S1600536811039092/ff2027Isup3.cdx

Supplementary material file. DOI: 10.1107/S1600536811039092/ff2027Isup4.cml

Additional supplementary materials:  crystallographic information; 3D view; checkCIF report

## Figures and Tables

**Fig. 1.**
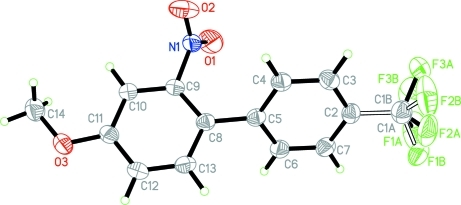
The structure of (I) with 50% probability displacement ellipsoids for non-hydrogen atoms showing the disordered –CF3 group.

## supplementary crystallographic information

### Experimental

To a solution of 4-bromo-trifluoromethylphenyl (5 mmol) and 4-methoxy-2-nitro-phenylboronic acid (6 mmol) in 20 ml water and 20 ml methanol was added Pd(OAc)_2_ (5 mmol) and K_2_CO_3_ (10 mmol). After stirring the reaction mixture for 12 h at room temperature, the aqueous phases were extracted with 100 ml ethyl acetate. The organic extracts were washed with 200 ml saturated aqueous sodium chloride, dried over sodium sulfate, filtered, and concentrated under reduced pressure. The resulting crude material was purified *via* silica gel chromatography (5% ethyl acetate/hexane) to afford a translucent solid in a yield of 80%. Crystals suitable for single-crystal X-ray diffraction were obtained by recrystallization from methanol at room temperature in a total yield of 32%. Analysis found: C 56.6, H 3.3, N 4.6%; C_14_H_10_F_3_NO_3_ requires: C 56.6, H 3.4, N 4.7%. _1_H NMR (400 MHz, CDCl_3_) 7.66 (d, *J* = 8.1 Hz, 2H), 7.45 (d, *J* = 2.6 Hz, 1H), 7.40 (d, *J* = 8.0 Hz, 2H), 7.32 (d, *J* = 8.5 Hz, 1H), 7.19 (dd, *J* = 8.6, 2.6 Hz, 1H), 3.92 (s, 3H).

### Refinement

All H-atoms were positioned geometrically and included in the refinement in the riding-model approximation, with *U*_iso_(H)= 1.5Ueq(methyl C) and 1.2Ueq(aromatic C). The –CF_3_ group is disordered over two sites with occupancies of 0.457 (8) and 0.543 (8). For this fragment, some anisotropic displacement ellipsoids were rather elongated which led us to use the ISOR restraints (Sheldrick, 2008).

### Crystal data

**Table:**

C_14_H_10_F_3_NO_3_	*F*(000) = 608
*M**_r_* = 297.23	*D*_x_ = 1.501 Mg m^−^^3^
Monoclinic, *P*2_1_/*c*	Mo *K*α radiation, λ = 0.71073 Å
Hall symbol: -P 2ybc	Cell parameters from 1880 reflections
*a* = 8.1956 (13) Å	θ = 2.8–22.6°
*b* = 20.777 (3) Å	µ = 0.13 mm^−^^1^
*c* = 7.9715 (12) Å	*T* = 293 K
β = 104.240 (2)°	Block, colorless
*V* = 1315.7 (3) Å^3^	0.26 × 0.24 × 0.20 mm
*Z* = 4	

### Data collection

**Table:**

Bruker APEXII CCD area-detector diffractometer	3235 independent reflections
Radiation source: fine-focus sealed tube	1910 reflections with *I* > 2σ(*I*)
graphite	*R*_int_ = 0.029
phi and ω scans	θ_max_ = 28.3°, θ_min_ = 2.8°
Absorption correction: multi-scan (*SADABS*; Sheldrick, 1996)	*h* = −10→10
*T*_min_ = 0.966, *T*_max_ = 0.974	*k* = −27→27
10512 measured reflections	*l* = −10→10

### Refinement

**Table:**

Refinement on *F*^2^	Secondary atom site location: difference Fourier map
Least-squares matrix: full	Hydrogen site location: inferred from neighbouring sites
*R*[*F*^2^ > 2σ(*F*^2^)] = 0.050	H atoms treated by a mixture of independent and constrained refinement
*wR*(*F*^2^) = 0.161	*w* = 1/[σ^2^(*F*_o_^2^) + (0.0741*P*)^2^ + 0.1504*P*] where *P* = (*F*_o_^2^ + 2*F*_c_^2^)/3
*S* = 1.05	(Δ/σ)_max_ = 0.001
3235 reflections	Δρ_max_ = 0.30 e Å^−^^3^
243 parameters	Δρ_min_ = −0.21 e Å^−^^3^
36 restraints	Extinction correction: *SHELXL*, Fc^*^=kFc[1+0.001xFc^2^λ^3^/sin(2θ)]^-1/4^
Primary atom site location: structure-invariant direct methods	Extinction coefficient: 0.026 (4)

### Special details

**Table:**

Geometry. All e.s.d.'s (except the e.s.d. in the dihedral angle between two l.s. planes) are estimated using the full covariance matrix. The cell e.s.d.'s are taken into account individually in the estimation of e.s.d.'s in distances, angles and torsion angles; correlations between e.s.d.'s in cell parameters are only used when they are defined by crystal symmetry. An approximate (isotropic) treatment of cell e.s.d.'s is used for estimating e.s.d.'s involving l.s. planes.
Refinement. Refinement of *F*^2^ against ALL reflections. The weighted *R*-factor *wR* and goodness of fit *S* are based on *F*^2^, conventional *R*-factors *R* are based on *F*, with *F* set to zero for negative *F*^2^. The threshold expression of *F*^2^ > σ(*F*^2^) is used only for calculating *R*-factors(gt) *etc*. and is not relevant to the choice of reflections for refinement. *R*-factors based on *F*^2^ are statistically about twice as large as those based on *F*, and *R*- factors based on ALL data will be even larger.

### Fractional atomic coordinates and isotropic or equivalent isotropic displacement parameters (Å^2^)

**Table:**

	*x*	*y*	*z*	*U*_iso_*/*U*_eq_	Occ. (<1)
C1A	0.6447 (4)	0.19880 (14)	0.4301 (4)	0.0705 (7)	0.46
F1A	0.6131 (8)	0.1395 (3)	0.3972 (18)	0.147 (3)	0.46
F2A	0.7487 (9)	0.2001 (4)	0.5832 (6)	0.108 (2)	0.46
F3A	0.7514 (8)	0.2162 (4)	0.3299 (10)	0.1111 (16)	0.46
C1B	0.6447 (4)	0.19880 (14)	0.4301 (4)	0.0705 (7)	0.54
F1B	0.6331 (8)	0.1469 (3)	0.5270 (8)	0.1152 (18)	0.54
F2B	0.7864 (6)	0.2248 (3)	0.4985 (14)	0.142 (2)	0.54
F3B	0.6443 (9)	0.1723 (3)	0.2844 (5)	0.1143 (16)	0.54
O3	−0.3255 (2)	0.48543 (8)	0.4265 (2)	0.0667 (5)	
N1	0.0999 (2)	0.42470 (11)	0.1294 (2)	0.0604 (5)	
C2	0.4994 (3)	0.24308 (11)	0.4159 (3)	0.0549 (6)	
C3	0.5169 (3)	0.30787 (12)	0.3869 (3)	0.0581 (6)	
C4	0.3843 (3)	0.34936 (11)	0.3798 (3)	0.0551 (6)	
C5	0.2308 (3)	0.32692 (10)	0.4005 (3)	0.0496 (5)	
C6	0.2145 (3)	0.26131 (11)	0.4274 (3)	0.0573 (6)	
C7	0.3473 (3)	0.21981 (12)	0.4348 (3)	0.0606 (6)	
C8	0.0882 (3)	0.37089 (10)	0.4025 (3)	0.0485 (5)	
C9	0.0257 (3)	0.41719 (10)	0.2790 (3)	0.0484 (5)	
C10	−0.1091 (3)	0.45702 (10)	0.2791 (3)	0.0504 (5)	
C11	−0.1881 (3)	0.45081 (10)	0.4134 (3)	0.0512 (5)	
C12	−0.1259 (3)	0.40693 (11)	0.5441 (3)	0.0574 (6)	
H12	−0.1751	0.4039	0.6372	0.069*	
C13	0.0072 (3)	0.36797 (11)	0.5379 (3)	0.0568 (6)	
C14	−0.4039 (3)	0.52531 (14)	0.2844 (4)	0.0754 (8)	
H14A	−0.3272	0.5585	0.2700	0.113*	
H14B	−0.5032	0.5445	0.3064	0.113*	
H14C	−0.4343	0.4998	0.1810	0.113*	
H10	−0.146 (3)	0.4878 (10)	0.188 (3)	0.053 (6)*	
H4	0.401 (3)	0.3937 (11)	0.364 (3)	0.064 (7)*	
H11	0.044 (3)	0.3381 (11)	0.630 (3)	0.063 (6)*	
H6	0.112 (3)	0.2454 (11)	0.442 (3)	0.062 (6)*	
H7	0.332 (3)	0.1735 (12)	0.454 (3)	0.067 (7)*	
H3	0.622 (3)	0.3236 (11)	0.371 (3)	0.068 (7)*	
O2	0.1707 (3)	0.47594 (11)	0.1180 (3)	0.0972 (7)	
O1	0.0856 (3)	0.38237 (10)	0.0266 (3)	0.1025 (8)	

### Atomic displacement parameters (Å^2^)

**Table:**

	*U*^11^	*U*^22^	*U*^33^	*U*^12^	*U*^13^	*U*^23^
C1A	0.0697 (18)	0.0762 (18)	0.0648 (17)	0.0077 (14)	0.0150 (13)	−0.0032 (14)
F1A	0.114 (4)	0.079 (3)	0.239 (7)	0.007 (3)	0.025 (6)	−0.054 (5)
F2A	0.099 (4)	0.151 (6)	0.063 (2)	0.062 (4)	−0.001 (2)	0.004 (3)
F3A	0.089 (3)	0.141 (4)	0.123 (4)	0.043 (3)	0.061 (3)	0.041 (3)
C1B	0.0697 (18)	0.0762 (18)	0.0648 (17)	0.0077 (14)	0.0150 (13)	−0.0032 (14)
F1B	0.126 (4)	0.116 (4)	0.115 (3)	0.066 (3)	0.053 (3)	0.055 (3)
F2B	0.065 (2)	0.122 (4)	0.221 (6)	0.017 (2)	0.002 (4)	−0.038 (4)
F3B	0.151 (4)	0.128 (3)	0.070 (2)	0.067 (3)	0.039 (2)	−0.007 (2)
O3	0.0589 (10)	0.0774 (11)	0.0701 (11)	0.0099 (8)	0.0280 (8)	0.0027 (8)
N1	0.0601 (12)	0.0727 (13)	0.0524 (11)	0.0114 (10)	0.0215 (9)	0.0146 (10)
C2	0.0553 (13)	0.0601 (13)	0.0466 (12)	0.0065 (11)	0.0076 (10)	−0.0014 (9)
C3	0.0517 (13)	0.0662 (15)	0.0568 (13)	−0.0055 (11)	0.0139 (10)	−0.0024 (11)
C4	0.0562 (14)	0.0512 (13)	0.0586 (13)	−0.0068 (11)	0.0152 (10)	0.0017 (10)
C5	0.0518 (12)	0.0518 (12)	0.0436 (11)	−0.0029 (10)	0.0085 (9)	0.0027 (9)
C6	0.0535 (14)	0.0536 (13)	0.0655 (14)	−0.0051 (11)	0.0161 (11)	0.0067 (10)
C7	0.0651 (15)	0.0524 (13)	0.0637 (14)	0.0005 (12)	0.0148 (11)	0.0058 (11)
C8	0.0477 (12)	0.0475 (11)	0.0501 (12)	−0.0066 (9)	0.0115 (9)	0.0023 (9)
C9	0.0518 (12)	0.0519 (11)	0.0438 (11)	−0.0031 (9)	0.0166 (9)	0.0019 (9)
C10	0.0531 (12)	0.0499 (11)	0.0501 (12)	0.0002 (10)	0.0161 (10)	0.0056 (10)
C11	0.0489 (12)	0.0529 (12)	0.0545 (12)	−0.0062 (10)	0.0176 (10)	−0.0032 (10)
C12	0.0590 (14)	0.0657 (14)	0.0529 (12)	−0.0066 (11)	0.0243 (10)	0.0026 (10)
C13	0.0611 (14)	0.0604 (13)	0.0492 (12)	−0.0040 (11)	0.0144 (10)	0.0104 (10)
C14	0.0611 (16)	0.0862 (18)	0.0803 (18)	0.0158 (14)	0.0201 (13)	0.0066 (14)
O2	0.1010 (15)	0.1191 (17)	0.0805 (14)	−0.0256 (13)	0.0393 (12)	0.0224 (12)
O1	0.149 (2)	0.0977 (15)	0.0789 (13)	0.0180 (14)	0.0628 (14)	−0.0116 (11)

### Geometric parameters (Å, °)

**Table:**

C1A—F1A	1.273 (6)	C5—C8	1.487 (3)
C1A—F2A	1.306 (6)	C6—C7	1.378 (3)
C1A—F3A	1.370 (5)	C6—H6	0.93 (2)
C1A—C2	1.487 (3)	C7—H7	0.99 (2)
O3—C11	1.361 (3)	C8—C9	1.381 (3)
O3—C14	1.423 (3)	C8—C13	1.401 (3)
N1—O1	1.188 (3)	C9—C10	1.381 (3)
N1—O2	1.226 (3)	C10—C11	1.387 (3)
N1—C9	1.474 (2)	C10—H10	0.96 (2)
C2—C3	1.379 (3)	C11—C12	1.384 (3)
C2—C7	1.380 (3)	C12—C13	1.369 (3)
C3—C4	1.377 (3)	C12—H12	0.9300
C3—H3	0.96 (2)	C13—H11	0.95 (2)
C4—C5	1.389 (3)	C14—H14A	0.9600
C4—H4	0.94 (2)	C14—H14B	0.9600
C5—C6	1.391 (3)	C14—H14C	0.9600
			
F1A—C1A—F2A	105.4 (6)	C6—C7—H7	119.2 (14)
F1A—C1A—F3A	105.2 (5)	C2—C7—H7	120.7 (14)
F2A—C1A—F3A	100.1 (5)	C9—C8—C13	114.61 (19)
F1A—C1A—C2	117.7 (4)	C9—C8—C5	125.07 (17)
F2A—C1A—C2	112.6 (3)	C13—C8—C5	120.31 (19)
F3A—C1A—C2	114.0 (3)	C10—C9—C8	124.99 (18)
C11—O3—C14	117.82 (17)	C10—C9—N1	115.23 (18)
O1—N1—O2	124.0 (2)	C8—C9—N1	119.73 (18)
O1—N1—C9	119.3 (2)	C9—C10—C11	118.1 (2)
O2—N1—C9	116.6 (2)	C9—C10—H10	120.4 (12)
C3—C2—C7	119.6 (2)	C11—C10—H10	121.5 (12)
C3—C2—C1A	120.2 (2)	O3—C11—C12	116.66 (18)
C7—C2—C1A	120.2 (2)	O3—C11—C10	124.2 (2)
C4—C3—C2	120.3 (2)	C12—C11—C10	119.1 (2)
C4—C3—H3	120.5 (14)	C13—C12—C11	120.76 (19)
C2—C3—H3	119.2 (14)	C13—C12—H12	119.6
C3—C4—C5	120.9 (2)	C11—C12—H12	119.6
C3—C4—H4	118.5 (15)	C12—C13—C8	122.4 (2)
C5—C4—H4	120.6 (15)	C12—C13—H11	117.6 (14)
C4—C5—C6	118.1 (2)	C8—C13—H11	120.1 (14)
C4—C5—C8	122.17 (19)	O3—C14—H14A	109.5
C6—C5—C8	119.66 (19)	O3—C14—H14B	109.5
C7—C6—C5	121.0 (2)	H14A—C14—H14B	109.5
C7—C6—H6	119.8 (14)	O3—C14—H14C	109.5
C5—C6—H6	119.2 (14)	H14A—C14—H14C	109.5
C6—C7—C2	120.1 (2)	H14B—C14—H14C	109.5
			
F1A—C1A—C2—C3	−156.3 (8)	C13—C8—C9—C10	−2.3 (3)
F2A—C1A—C2—C3	80.8 (6)	C5—C8—C9—C10	178.5 (2)
F3A—C1A—C2—C3	−32.4 (6)	C13—C8—C9—N1	−179.6 (2)
F1A—C1A—C2—C7	24.9 (8)	C5—C8—C9—N1	1.2 (3)
F2A—C1A—C2—C7	−98.0 (6)	O1—N1—C9—C10	−111.7 (2)
F3A—C1A—C2—C7	148.7 (5)	O2—N1—C9—C10	66.8 (3)
C7—C2—C3—C4	1.1 (3)	O1—N1—C9—C8	65.8 (3)
C1A—C2—C3—C4	−177.7 (2)	O2—N1—C9—C8	−115.6 (2)
C2—C3—C4—C5	−0.4 (3)	C8—C9—C10—C11	0.5 (3)
C3—C4—C5—C6	−0.4 (3)	N1—C9—C10—C11	177.87 (19)
C3—C4—C5—C8	176.8 (2)	C14—O3—C11—C12	−172.8 (2)
C4—C5—C6—C7	0.5 (3)	C14—O3—C11—C10	7.2 (3)
C8—C5—C6—C7	−176.7 (2)	C9—C10—C11—O3	−177.8 (2)
C5—C6—C7—C2	0.2 (4)	C9—C10—C11—C12	2.2 (3)
C3—C2—C7—C6	−1.0 (3)	O3—C11—C12—C13	177.1 (2)
C1A—C2—C7—C6	177.8 (2)	C10—C11—C12—C13	−2.9 (3)
C4—C5—C8—C9	50.9 (3)	C11—C12—C13—C8	0.9 (4)
C6—C5—C8—C9	−131.9 (2)	C9—C8—C13—C12	1.6 (3)
C4—C5—C8—C13	−128.2 (2)	C5—C8—C13—C12	−179.2 (2)
C6—C5—C8—C13	48.9 (3)		
